# Meningococcal Purpura Fulminans: A Rare Presentation in an Adult Case of Serogroup W135 Infection

**DOI:** 10.7759/cureus.94253

**Published:** 2025-10-09

**Authors:** Usamah Al-Anbagi, Muayad K Ahmad, Mohamed G Mohamedali, Abdulrahman Saad, Marwa I Elaziz, Abdulqadir J Nashwan, Hatem M Abusriwil

**Affiliations:** 1 Internal Medicine, Hazm Mebaireek General Hospital/Hamad Medical Corporation, Doha, QAT; 2 Internal Medicine, Hamad Medical Corporation, Doha, QAT; 3 Medicine, Ministry of Public Health, Doha, QAT; 4 Medical Education, Hamad Medical Corporation, Doha, QAT; 5 Nursing and Midwifery Research, Hamad Medical Corporation, Doha, QAT

**Keywords:** case report, disseminated intravascular coagulopathy (dic), meningococcal purpura fulminans (mpf), neisseria meningitidis, serogroup w135

## Abstract

Meningococcal purpura fulminans is a rare but serious complication of invasive *Neisseria meningitidis* infection, marked by widespread clotting problems and skin tissue death. Diagnosis can be tricky, especially when patients show unusual early symptoms that do not fit the classic pattern. We present an adult case infected with serogroup W135 who initially had non-specific symptoms such as sore throat, which progressed quickly to severe septic shock and purpura fulminans. The patient required intensive care and tailored treatments, including antibiotics and supportive care. This case highlights how varied the presentation can be and stresses the need for early recognition and aggressive management to improve outcomes and prevent lasting damage.

## Introduction

Meningococcal disease is a major public health concern worldwide, associated with high morbidity and mortality, particularly in children and young adults. *Neisseria meningitidis*, a Gram-negative diplococcus, is responsible for invasive infections that may present as meningitis or fulminant septicemia, often leading to devastating complications, including shock, disseminated intravascular coagulation (DIC), and purpura fulminans [1-3]. Outbreaks and sporadic cases continue to occur globally, with epidemiological variations across regions and serogroups [4,5].

Purpura fulminans is a rare but life-threatening complication of severe sepsis, characterized by rapidly progressive purpuric skin lesions, vascular thrombosis, and DIC [6,7]. It results from dysregulation of the coagulation system, often related to deficiencies in natural anticoagulant pathways, such as protein C, protein S, or antithrombin III [8-11]. Clinically, it manifests with hemorrhagic infarction of the skin and may progress to extensive necrosis requiring surgical intervention or even limb amputation [12,13].

Although purpura fulminans has been described in association with several infectious agents, meningococcal septicemia remains one of the most important causes, and timely recognition is critical to reduce mortality [10]. We present a case of meningococcal septicemia complicated by purpura fulminans in an adult patient.

## Case presentation

History

A previously healthy 36-year-old Indian man presented with a four-day history of gradual onset of fever, headache, generalized body aches, sore throat, and a noticeable change in voice. Over the preceding two days prior to admission, he developed difficulty swallowing along with bilateral upper neck pain. He denied cough, shortness of breath, chest pain, abdominal pain, vomiting, diarrhea, dysuria, skin rash, or recent travel. Family history was unremarkable. The patient denied any recent use of herbal, illicit, or over-the-counter medications. He is a non-smoker. There was no history of contact with individuals who were sick or had a prior history of similar illnesses.

Examination

On examination, he was conscious, alert, and oriented, with a Glasgow Coma Scale 14 of 15 (eye opening: 4/4, verbal response: 4/5, motor response: 6/6) [14], but appeared dehydrated, irritable, and hypotensive with a blood pressure of 101/39 mmHg, tachycardia at 133 bpm, respiratory rate of 22, temperature of 38.7 °C, and oxygen saturation of 97% on room air. His pharynx was markedly congested with enlarged tonsils nearly obstructing the airway. Chest examination revealed good bilateral air entry with normal breath sounds, and cardiovascular examination showed normal heart sounds with no murmurs or added sounds. The abdomen was soft, non-distended, and non-tender, with no organomegaly or guarding. There was no costovertebral angle tenderness, peripheral edema, or meningeal signs. Skin examination revealed no rash, petechiae, or purpura at presentation. Cranial nerves and motor-sensory examinations were intact, and there was no focal neurological deficit.

Investigation and treatment

Laboratory workup revealed leukocytosis, thrombocytopenia, elevated creatinine, low potassium levels, metabolic acidosis, markedly elevated inflammatory markers, deranged liver enzymes, and very high CK, myoglobin, and DIC parameters (Table 1).

**Table 1:** Patient laboratory values ALP: Alkaline Phosphatase; ALT: Alanine Aminotransferase; APTT: Activated Partial Thromboplastin Time; AST: Aspartate Aminotransferase; CK: Creatine Kinase; CRP: C-Reactive Protein; DD: D-dimer; Hb: Hemoglobin; INR: International Normalized Ratio; K⁺: Potassium; Na⁺: Sodium; PCT: Procalcitonin; Plt: Platelets; PT: Prothrombin Time; WBC: White Blood Cells

Parameter	On Admission	On Discharge	Normal Values
WBC	18.3	7.5	6.2 × 10³/μL
Hb	16.1	12.1	13-17 g/dL
Plt	33	294	150-410 × 10³/μL
CRP	179	14.4	0-5 mg/L
PCT	>100	0.7	< 0.05 ng/mL
Urea	5.8	3.5	2.5-7.8 mmol/L
Creatinine	255	55	62-106 μmol/L
K⁺	2.8	4.0	3.5-5.3 mmol/L
Na⁺	131	129	133-146 mmol/L
Total Protein	60	87	60-80 g/L
Albumin	31	30	35-50 g/L
ALT	67	58	0-41 IU/L
AST	58	44	0-41 IU/L
ALP	168	199	40-129 U/L
Bilirubin	38	17	0-21 μmol/L
PT	22.9	15.0	9.4-12.5 s
INR	2.0	1.4	< 1.0
APTT	64.4	109.0	25.1-36.5 s
DD	125.9	Not tested	0-0.5 mg/L FEU*
Troponin	20	Not tested	3-15 ng/L
CK	3025	47	39-308 U/L
Myoglobin	3405	Not tested	28-72 ng/mL
Lactatic acid	13.4	Not tested	0.5-2.2 mmol/L

**Figure 1:**
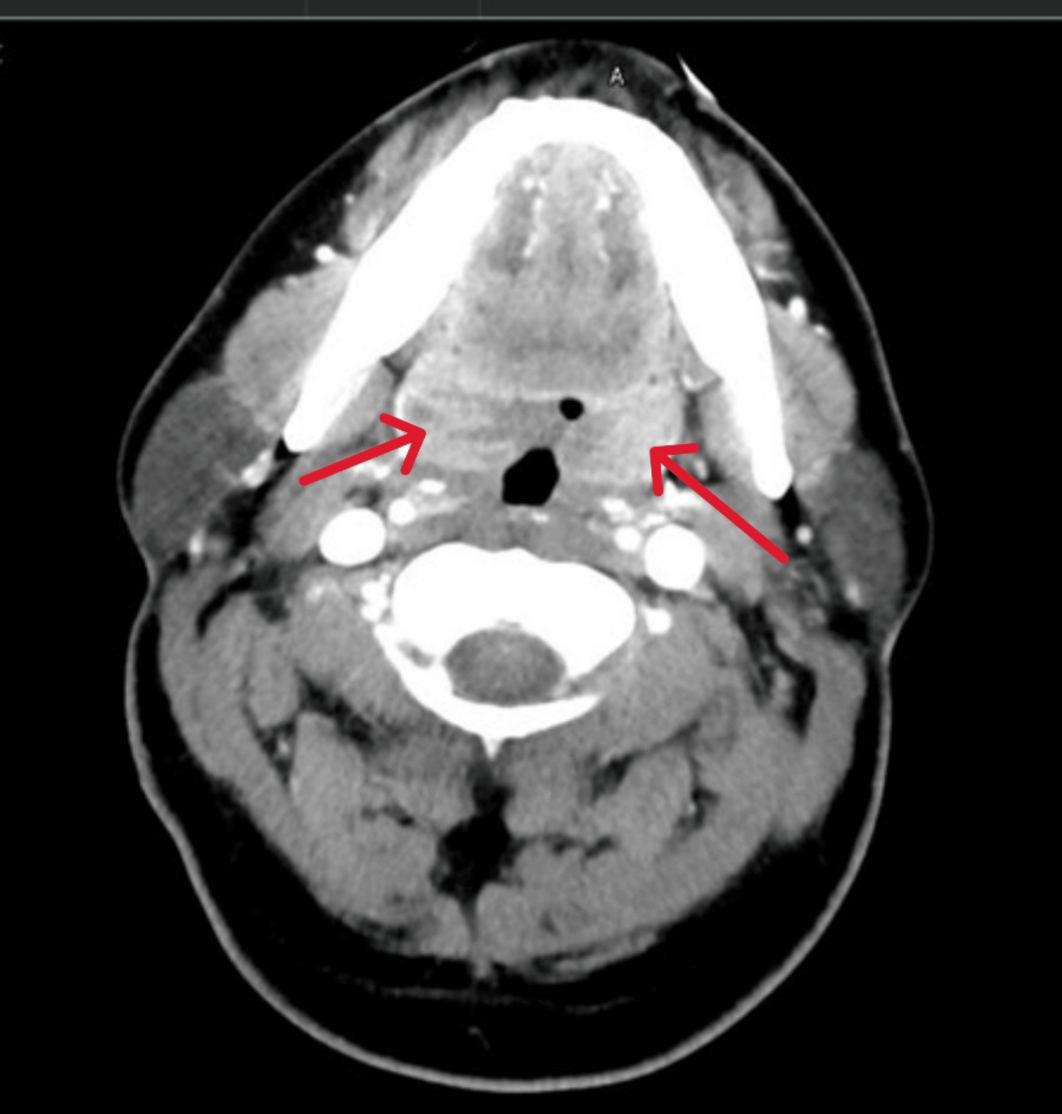
CT neck, axial view, revealed diffuse tonsillar enhancement and enlargement (red arrows) CT: Computerized tomography

**Figure 2:**
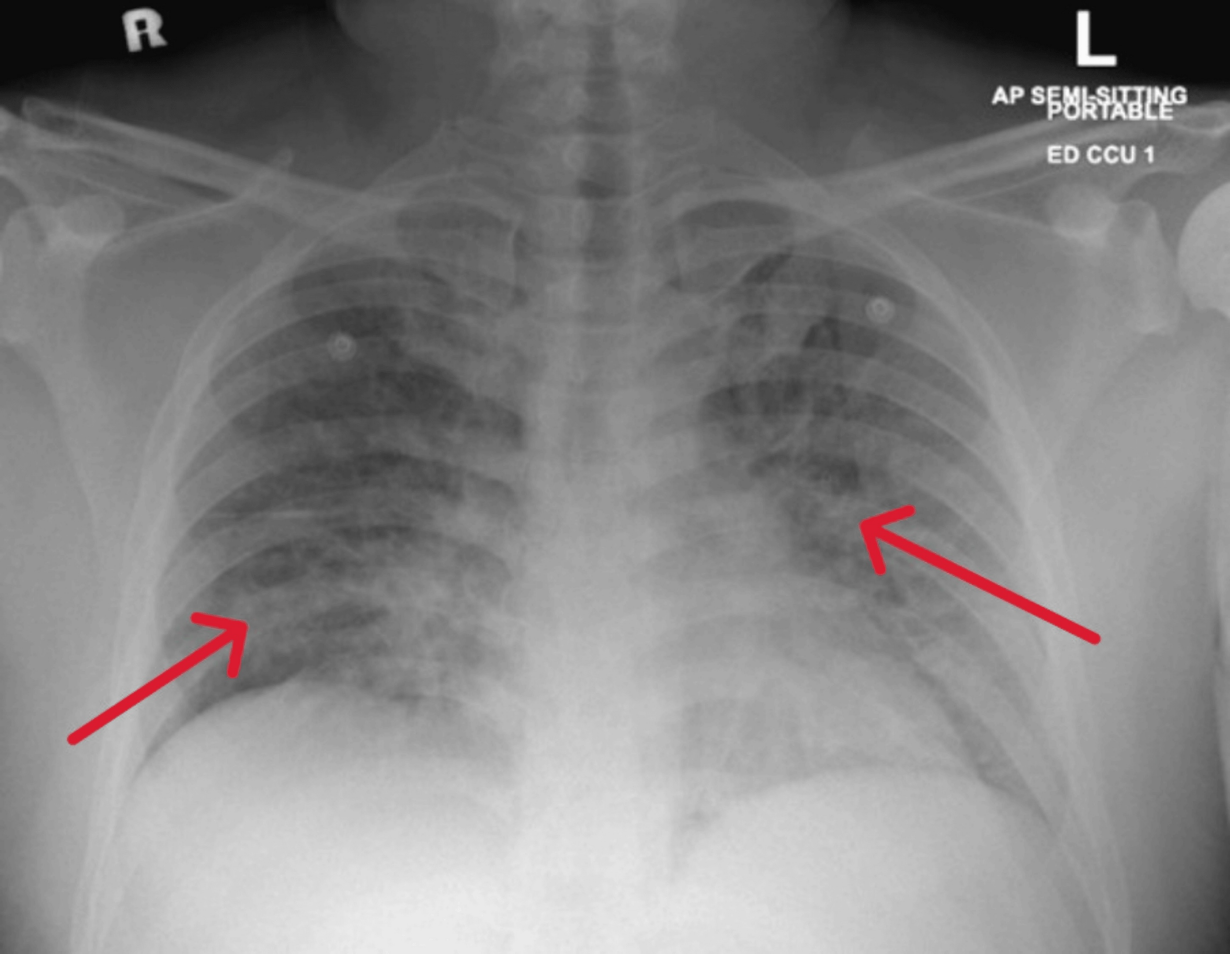
Chest X-ray, PA view, revealed bilateral infiltration (red arrows) PA: posteroanterior

CT of the neck showed diffuse adenoidal and tonsillar enlargement without abscess formation (Figure 1), and chest X-ray revealed bilateral infiltrates (Figure 2).

**Figure 3:**
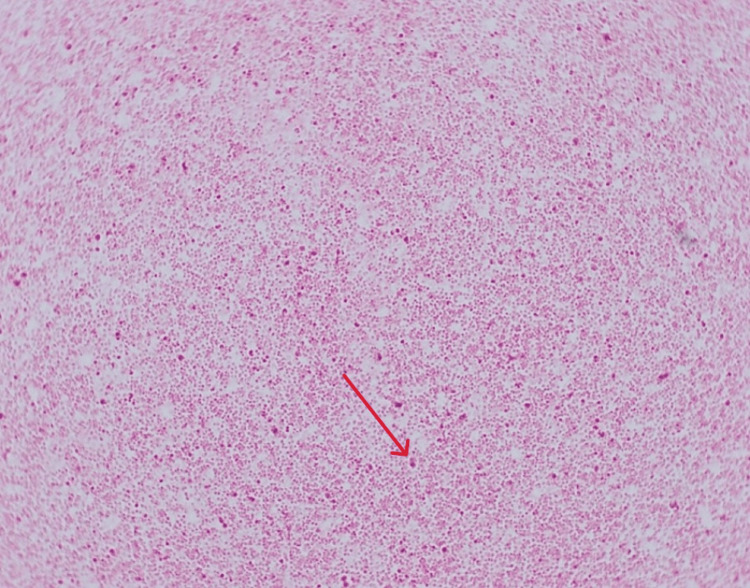
Gram-negative diplococci observed on direct Gram stain of a positive blood culture (red arrow)

He was admitted as a case of tonsillitis with sepsis, but soon deteriorated, requiring vasopressors with noradrenaline. He became drowsy with declining consciousness and was subsequently intubated. Blood cultures returned positive for *N. meningitidis *serogroup W135 (Figure 3), leading to a revised diagnosis of meningococcemia with meningitis, septic shock, rhabdomyolysis, and acute kidney injury.

**Figure 4:**
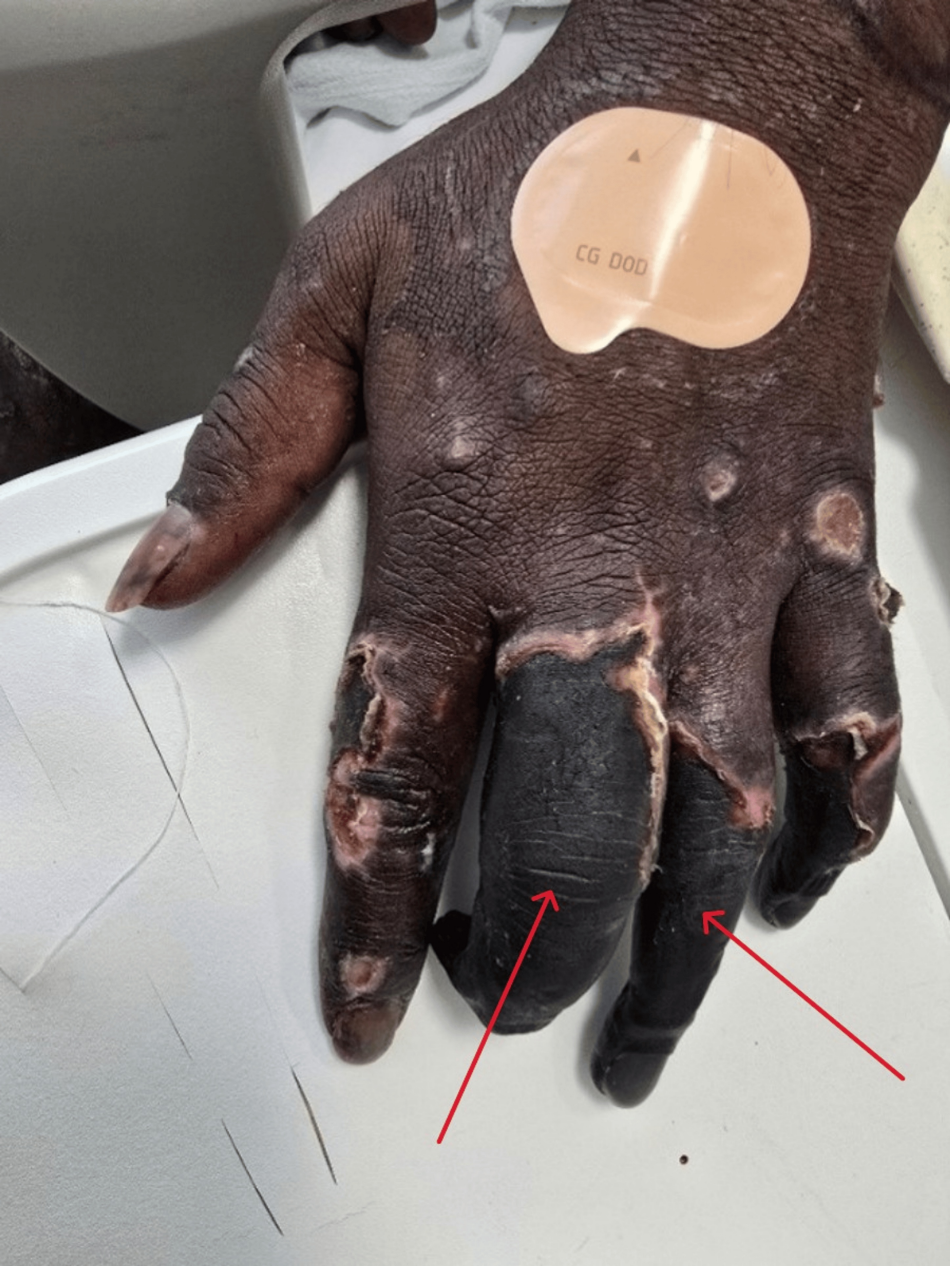
Areas of necrosis, ulceration, and black discoloration affecting primarily the middle and ring fingers, consistent with purpura fulminans (red arrows)

**Figure 5:**
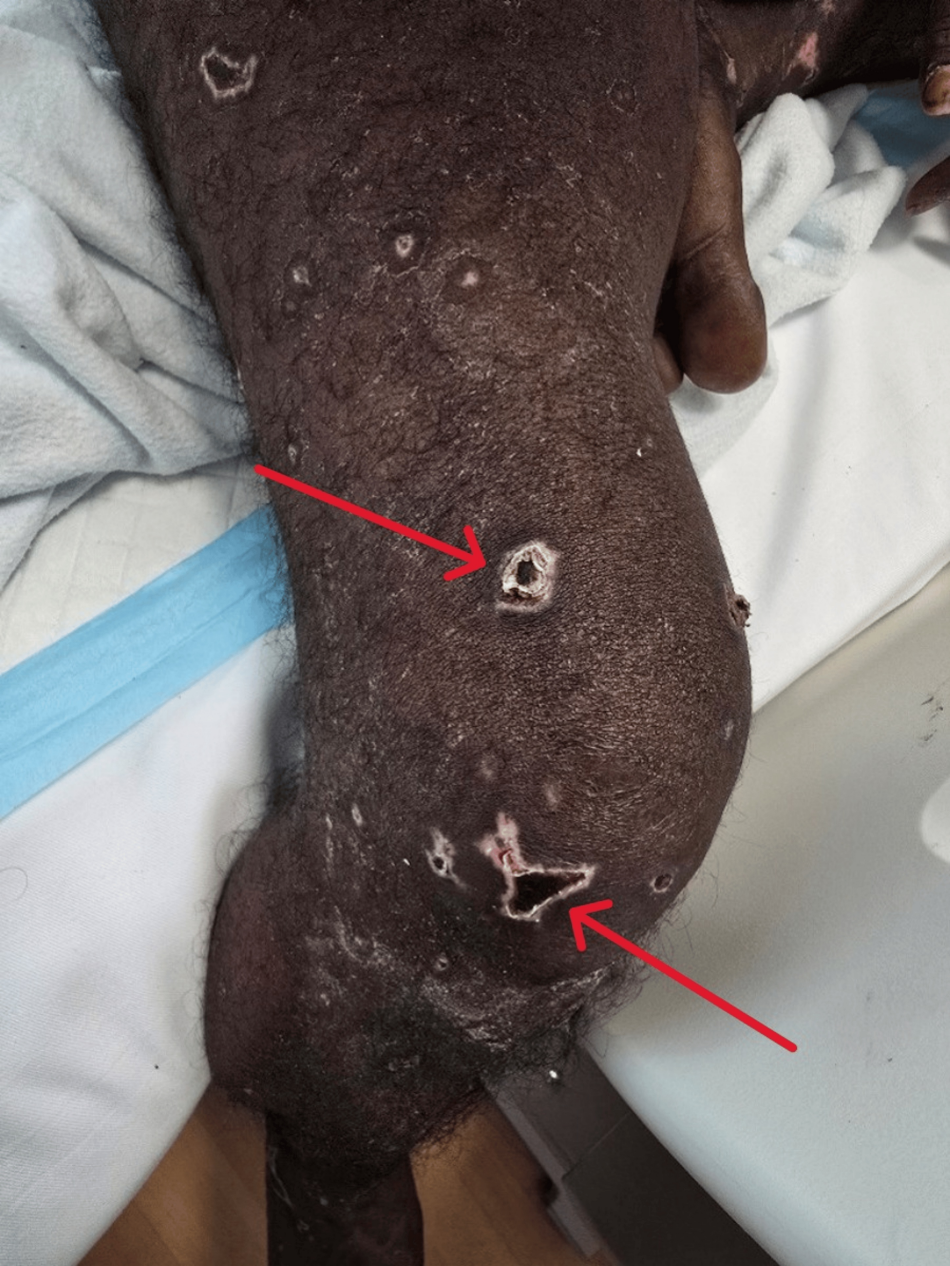
Multiple well-demarcated necrotic ulcers with dark eschar centers and erythematous-violaceous borders, consistent with purpura fulminans (red arrows)

Ceftriaxone was escalated to 2 g twice daily for 10 days, as per infectious disease advice. Despite improving labs and clearance of bacteremia, he remained critically ill on mechanical ventilation and vasopressors, prompting escalation to meropenem. He was extubated after five days, but by day four developed small necrotic lesions on his hand and foot (Figure 4), rapidly progressing to gangrene involving fingers, toes, arms, legs, buttocks, and perioral regions (Figure 5).

Antibiotics were later (after stabilization) de-escalated to ceftriaxone, and wound care with topical glyceryl trinitrate was initiated under the guidance of vascular and dermatology specialists. He was completely hemodynamically stable, with vital signs and blood pressure maintained within normal limits. The patient remained afebrile, with oxygen saturation maintained on room air. He was mobile, fully conscious, and oriented. Despite progressive gangrene, his overall condition stabilized. After a 30-day hospitalization, he opted to discharge himself against medical advice to return to his home country for continued rehabilitation.

## Discussion

*N. meningitidis* remains a leading cause of bacterial meningitis and septicemia worldwide, disproportionately affecting children and young adults. In the United States, the annual incidence ranges from 0.5 to 1.5 per 100,000, with serogroups B, C, and Y predominating, while serogroup W135 has caused notable outbreaks associated with travel to the Middle East [1]. Mortality still hovers around 10-15% even in well-resourced settings, largely due to the organism’s propensity for rapid progression to septic shock and multiorgan failure [2]. This case illustrates the often deceptive clinical spectrum of invasive meningococcal disease, particularly when it presents with upper respiratory symptoms instead of the classic triad of fever, rash, and meningismus. The patient began with pharyngitis, odynophagia, and voice change, misleading clinicians toward a local ENT infection, when in fact it heralded meningococcemia. These atypical features can contribute to diagnostic delays that prove fatal without early empiric therapy [3-5].

Purpura fulminans is a rare but catastrophic complication characterized by DIC with extensive microvascular thrombosis and hemorrhagic skin necrosis. It typically presents as rapidly evolving retiform purpura with irregular, branched, or angular lesions (6), as was evident in this case. This syndrome is generally classified into hereditary and acquired forms. The hereditary type most often results from homozygous protein C deficiency, typically manifesting in neonates with severe purpura and thrombosis, while heterozygous individuals usually develop venous thromboembolism without DIC [6]. The acquired form is frequently linked to overwhelming infections such as meningococcemia, where a strong inflammatory response drives excessive tissue factor expression and the release of procoagulant microparticles, leading to widespread coagulation activation [6].

Clinically, purpura fulminans is recognized by its abrupt onset of thrombotic DIC with characteristic skin findings, and while biopsy is seldom required, it can sometimes be performed to confirm the diagnosis [6]. Regardless of cause, purpura fulminans follows a shared pathway involving reduced protein C levels or activity, which promotes unchecked clot formation [7]. In severe sepsis, the widespread inflammatory response activates coagulation and complement systems and damages the endothelium. Cytokines such as interleukin-1 and TNF-alpha suppress the production of protein C, protein S, and antithrombin while boosting prothrombotic factors such as factor VIII and fibrinogen [8]. Normally, activated protein C helps regulate clotting and inflammation by stabilizing the endothelium and downregulating pro-inflammatory signals, but its depletion in sepsis exacerbates microvascular thrombosis. This leads to consumption of platelets and clotting factors, paradoxically increasing bleeding risk [8]. Early skin lesions show small vessel blockage and capillary congestion; later stages demonstrate irreversible endothelial damage, dermal hemorrhage, and necrosis [9]. Ultimately, the loss of natural anticoagulants fosters more clotting, impairs fibrinolysis, and sustains inflammation [10].

Early recognition of sepsis-associated purpura fulminans is crucial, as prompt intervention can sometimes halt progression [4,11]. Management centers on rapidly controlling infection with antibiotics, supporting circulation and oxygenation, and correcting coagulation imbalances. Fresh frozen plasma or protein C concentrate is used to restore depleted clotting regulators [12]. Due to protein C’s short half-life, repeated doses are often needed. When bleeding is significant, platelet or cryoprecipitate transfusions may also be required [11]. Unfortunately, once tissue necrosis is established, surgical interventions such as debridement, fasciotomy, or amputation frequently become necessary [13].

Purpura fulminans lesions often advance within 24-48 hours to deep skin or soft-tissue necrosis, requiring weeks to heal and commonly leaving extensive scarring [13]. Without timely treatment, these areas can become gangrenous, sometimes necessitating limb amputation. The condition often involves microvascular thrombosis in organs such as the lungs, kidneys, brain, and adrenal glands, driving multi-organ failure, high early mortality, and long-term disability [13]. Inherited cases with protein C deficiency are prone to recurrent thrombotic events, and surviving infants may suffer neurological or visual impairment. New lesions may continue to appear for up to two weeks in post-infectious cases [15].

## Conclusions

In conclusion, this case highlights the wide-ranging and sometimes misleading presentations of meningococcal disease, from pharyngeal symptoms to severe purpura fulminans. It underscores the importance of maintaining a high level of clinical suspicion, initiating prompt broad-spectrum antibiotic therapy, and managing complications aggressively to improve outcomes. Moreover, this case serves as a reminder of the critical role of vaccination and robust public health strategies in preventing this potentially fatal yet largely avoidable infection.
